# Resting-State Networks of Awake Adolescent and Adult Squirrel Monkeys Using Ultra-High Field (9.4 T) Functional Magnetic Resonance Imaging

**DOI:** 10.1523/ENEURO.0173-23.2024

**Published:** 2024-05-03

**Authors:** Walid Yassin, Fernando B. de Moura, Sarah L. Withey, Lei Cao, Brian D. Kangas, Jack Bergman, Stephen J. Kohut

**Affiliations:** ^1^Behavioral Neuroimaging Laboratory, McLean Hospital, Belmont, Massachusetts 02478; ^2^Behavioral Biology Program, McLean Hospital, Belmont, Massachusetts 02478; ^3^Department of Psychiatry, Harvard Medical School, Boston, Massachusetts 02478; ^4^McLean Imaging Center, McLean Hospital, Belmont, Massachusetts 02478

**Keywords:** functional magnetic resonance imaging, neuroimaging, nonhuman primate, resting-state networks, translational neuroimaging, ultra-high field

## Abstract

Resting-state networks (RSNs) are increasingly forwarded as candidate biomarkers for neuropsychiatric disorders. Such biomarkers may provide objective measures for evaluating novel therapeutic interventions in nonhuman primates often used in translational neuroimaging research. This study aimed to characterize the RSNs of awake squirrel monkeys and compare the characteristics of those networks in adolescent and adult subjects. Twenty-seven squirrel monkeys [*n *= 12 adolescents (6 male/6 female) ∼2.5 years and *n *= 15 adults (7 male/8 female) ∼9.5 years] were gradually acclimated to awake scanning procedures; whole-brain fMRI images were acquired with a 9.4 T scanner. Group-level independent component analysis (ICA; 30 ICs) with dual regression was used to detect and compare RSNs. Twenty ICs corresponding to physiologically meaningful networks representing a range of neural functions, including motor, sensory, reward, and cognitive processes, were identified in both adolescent and adult monkeys. The reproducibility of these RSNs was evaluated across several ICA model orders. Adults showed a trend for greater connectivity compared with adolescent subjects in two of the networks of interest: (1) in the right occipital region with the OFC network and (2) in the left temporal cortex, bilateral occipital cortex, and cerebellum with the posterior cingulate network. However, when age was entered into the above model, this trend for significance was lost. These results demonstrate that squirrel monkey RSNs are stable and consistent with RSNs previously identified in humans, rodents, and other nonhuman primate species. These data also identify several networks in adolescence that are conserved and others that may change into adulthood.

## Significance Statement

Functional magnetic resonance imaging procedures have revealed important information about how the brain is modified by experimental manipulations, disease states, and aging throughout the lifespan. Preclinical neuroimaging, especially in nonhuman primates, has become a frequently used means to answer targeted questions related to brain resting-state functional connectivity. The present study characterized resting-state networks (RSNs) in adult and adolescent squirrel monkeys; 20 RSNs corresponding to networks representing a range of neural functions were identified. The RSNs identified here can be utilized in future studies examining the effects of experimental manipulations on brain connectivity in squirrel monkeys. These data also may be useful for comparative analysis with other primate species to provide an evolutionary perspective for understanding brain function and organization.

## Introduction

Resting-state fMRI (rsfMRI) is a technique used to map brain regional interactions inferred from the degree of temporal correlations between spontaneous low-frequency fluctuations (LFFs) in blood oxygenation level-dependent (BOLD) signal occurring in the absence of an explicit task (Friston, 1994; Biswal et al., 1995). rsfMRI can provide valuable information about the brain in a relatively short scan session and, unlike other functional neuroimaging techniques, does not require sustained attention or task performance. Investigations using rsfMRI have revealed multiple large-scale brain functional resting-state networks (RSNs; Biswal et al., 1995; Biswal, 2012) that are thought to subserve a variety of behavioral and cognitive domains. A hallmark of this approach is the high reproducibility of RSNs both within and between subjects—whether using seed-based (Vincent et al., 2007; Margulies et al., 2009; Babapoor-Farrokhran et al., 2013) or independent component analyses (ICAs; Damoiseaux et al., 2006, Moeller et al., 2009; Pendse et al., 2011). The stability and reproducibility of RSNs thus merit their use as candidate biomarkers for neurological and psychiatric disorders and, in fact, alterations in RSNs have been identified in a variety of psychiatric conditions (Bluhm et al., 2007; M. D. Greicius et al., 2007; Auer, 2008; M. Greicius, 2008; Yamada et al., 2017; Canario et al., 2021).

Many of the core functional networks that have been identified in humans (Biswal et al., 1995; Biswal, 2012) also have been identified in laboratory animal species (Smith et al., 2009, 2013) including nonhuman primates (NHPs; Hutchison et al., 2011; Belcher et al., 2013; Yacoub et al., 2020; C. Liu et al., 2021) and rodents (Jonckers et al., 2011; Lu et al., 2012). As a result, preclinical neuroimaging has become a frequently used means to answer targeted questions related to RSNs. NHPs, in particular, are an invaluable asset to this type of investigation. In addition to their genetic, phylogenetic, pharmacokinetic, and neurobiological similarity to humans (Weerts et al., 2007; Hutchison and Everling, 2012; Royo et al., 2021), NHPs with known pharmacological and behavioral histories can be studied longitudinally under carefully controlled conditions that are rarely, if ever, possible in humans.

To date, the majority of preclinical fMRI investigations have involved macaques, which have a long history of serving as subjects in behavioral, pharmacological, and neuroscience research, or marmosets, which are small enough to serve as subjects in ultra-high field scanners that tend to have relatively small bore size. However, squirrel monkeys provide an appealing alternative, highly accessible NHP species for neuroimaging studies: like macaques, squirrel monkeys have been widely used in many types of in vivo research (Abee, 1989; Royo et al., 2021) and, like marmosets, subjects are small enough (often <1 kg) for ultra-high field scanning. Importantly, when compared with marmosets, squirrel monkeys have larger brains with more pronounced gyrification [Royo et al. (2021), Fig. 1], and their performance on cognitive tasks align more closely with that of macaques and humans than other primate species (reviewed by Royo et al., 2021; see also Williams and Glasgow, 2000) highlighting the translational value of neuroscience research using squirrel monkey subjects. Despite their value and popularity in biomedical research, few MRI studies have been conducted in squirrel monkeys and, in particular, little is known regarding their RSNs.

The present study sought to provide a comprehensive characterization of RSNs in experimentally naive, awake squirrel monkeys using rsfMRI conducted at ultra-high field (9.4 T). Functional neuroimaging in awake subjects avoids the potential confounding influence of anesthetic agents on functional connectivity (Lv et al., 2016; Wu et al., 2016; Zhang et al., 2019) and enhances the translational value of such data. To accomplish this, a large cohort of adolescent and adult male and female squirrel monkeys initially were acclimated to awake scanning procedures at ultra-high field. The robustness of identified RSNs was then evaluated in squirrel monkeys and, as well, determined whether core networks differ as a function of age, that is, between adolescent and adult subjects.

## Materials and Methods

### Subjects

Twenty-seven squirrel monkeys (*Saimiri sciureus*) served as subjects and comprised two groups: adolescents that were ∼2.5 years of age (mean, 32.31 ± 0.35 months; *n *= 12; 6 male/6 female) and adults that were between 9 and 10 years of age (mean, 117.29 ± 0.74 months; *n *= 15; 7 male/8 female). Subjects were housed in a temperature- and humidity-controlled vivarium with a 12 h light/dark cycle (07:00–19:00). Monkeys had unlimited access to water in the home cage and were maintained at approximate *ad libitum* feeding weights with a nutritionally balanced diet of high protein chow (Purina Monkey Chow). Fresh fruit and vitamins were provided as part of a comprehensive environmental enrichment plan. The experimental protocol was approved by the Institutional Animal Care and Use Committee at McLean Hospital in a facility licensed by the US Department of Agriculture and conducted in accordance with guidelines provided by the Committee on Care and Use of Laboratory Animals of the Institute of Laboratory Animals Resources, Commission on Life Sciences.

### Behavioral training and acclimation for imaging procedures

**Figure 1.**
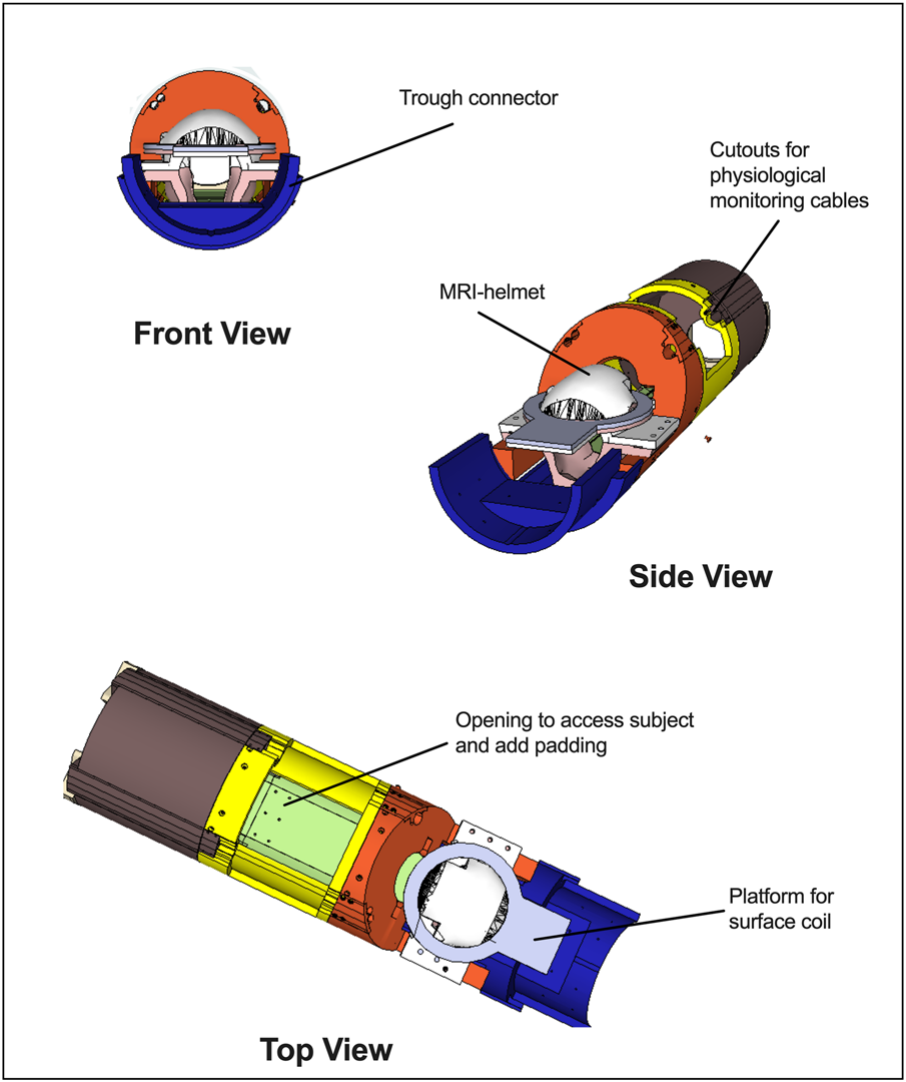
Schematic of the MRI apparatus developed for squirrel monkeys. Details about acclimation procedures are described in text.

Extensive behavioral training was used to acclimate subjects to the MR procedures which included a stepwise process of gradual progression through each of several stages of acclimation. Acclimation to the MRI apparatus typically occurred 5 days per week. Subjects were closely monitored throughout the acclimation process by evaluating resting heart rate as well as observations of subject behavior before and after training sessions. Using this extensive process, all subjects were successfully trained for awake MRI with no indication of behavioral or physiological changes before or after daily sessions. Initially, subjects were trained to rest in a prone position on their haunches within a custom-designed 3D printed (ABS plastic) chair (Fig. 1) enclosure for brief (5–10 min) sessions during which they were given access to intermittent presentations of ∼0.1 ml of 30% sweetened condensed milk by syringe. The duration of acclimation training was systematically increased over several days to 30 min, after which acclimation to the helmet began. The helmet was intended to fit comfortably and securely; its design was based on squirrel monkey anatomic images collected on McLean Hospital's 9.4 T Varian system. The helmet was 3D-printed (ABS plastic) and lined with padding to limit motion and optimize comfort for the subjects; several variations of the helmet that differ only in size were tailored with padded lining for each individual subject. The helmet, which also included a platform to position a transmit/receive surface coil, was mounted to the chair body with plastic screws (shown in Fig. 1). Once the subjects were acclimated to the helmet, the session duration was gradually extended to 60 min. The final phase of acclimation involved moving chaired subjects into an MRI simulator bore housed within the laboratory. During MRI simulation sessions, recorded sounds from the scanner were played at decibels similar to those within the actual scanner (∼90–100 dB). Vital signs (e.g., heart rate and oxygen saturation (SPO_2_); Nonin Model 7500FO) were tracked and recorded at 5 min intervals throughout both training and MR sessions and, using live video-feeds, subjects were continuously monitored by a trained research assistant (VID-CAM-MONO-1 with SOF-842, Med-Associates; 12M camera, MRC Systems). Normative ranges were 180–380 bpm for heart rate and 94–100% SPO_2_; these values were established in consultation with the McLean Hospital Attending Veterinarian in both unrestrained and restrained subjects and were never exceeded in any scan.

### MRI data acquisition

Images were acquired with a 9.4 T/400 mm diameter MR system (Varian Direct Drive, Varian). The system has a 116 mm inner diameter gradient with a maximum strength of 45 G/cm; a single loop head-encompassing transmit–receive RF coil was used. Preparatory scans included automated image-based shimming <75 Hz. fMRI images were acquired with a whole-brain gradient-echo planar imaging (EPI) sequence with TE, 8 ms; TR, 1,500 ms; flip angle, 90°; and voxel size, 0.99 × 0.99 × 0.93 mm; scan matrix was 64 on a 64 mm field of view (FOV) with 54 × 1 mm slices; and scan time for 1,200 volumes was 30 min. Distortion-matched anatomic images were acquired with a spin-echo EPI sequence (TE, 17.5 ms; TR, 1,500 ms; flip angle, 90°; averages, 8; scan matrix, 64 on a 64 mm FOV with 54 × 1 mm coronal slices matched to the fMRI slices).

### MRI data processing

The data were visually checked slice by slice for artifacts using all three orthogonal directions. Quality control was evaluated using MRI Quality Control tool (MRIQC; Esteban et al., 2017), and group mean motion statistics were calculated for the dataset. Intensity spiking was analyzed and resolved with an in-house program, “spikefix,” which is designed to find and remove spikes from fMRI datasets (https://github.com/bbfrederick/spikefix) with a threshold of 1.0 mm framewise displacement. FMRIB's Software Library (FSL, Oxford University) image preprocessing pipeline was used to process the data as follows (Smith et al., 2004; Jenkinson et al., 2012): the first 10 volumes from each scan were removed to allow for data stabilization. MCFLIRT tool (Jenkinson et al., 2002) in FSL was used for head motion correction by volume realignment to the middle volume. The resulting 12-motion correction positions were used as nuisance regressors. Session-averaged functional volumes were aligned to the VALiDATe (Schilling et al., 2017) T1w template through a 12 DOF affine transformation followed by adjustment of nonlinear distortion fields using the jip analysis toolkit (www.nitrc.org/projects/jip). Spatial smoothing was conducted using a Gaussian kernel of 2.0 mm FWHM. Registration to template space was carried out using jip. Temporal filtering was applied using a high-pass filter with a 100 s cutoff (0.01 Hz).

### ICA

Group-level independent component (gIC, Melodic, FSL) analysis was used to examine the resting-state fMRI data. The number of independent components (ICA 30) was chosen based on previously published methods in NHPs (Lu et al., 2012; Belcher et al., 2013). The ICA method was utilized as the primary analytic strategy for this study because it has been used across a variety of species and is a highly replicable, well-validated method for investigating large-scale brain networks using rsfMRI. The widespread use and acceptance of this approach permits the ICA maps obtained here in squirrel monkeys to be directly compared with those obtained in both human and other nonhuman subjects. The robustness of the identified ICA maps was examined using exploratory analysis of 20, 25, 35, and 40 model-specified components (Belcher et al., 2013). Hand classification (cf. Griffanti et al., 2017) was used to remove noise ICs with any of the following characteristics: (1) large number of small clusters, (2) cluster peaks in WM/CSF, (3) indiscriminate overlap with non-GM tissue, (4) ring-like stripes near the edge of the FOV, (5) localized to regions of air-tissue interface, or (6) streaks along the phase encoding direction. The physiologically relevant components were visually identified and most determined to be consistent with previously reported networks in humans, rhesus monkeys, marmosets, rats, and mice (Beckmann et al., 2005; Hutchison et al., 2011; Jonckers et al., 2011; Lu et al., 2012; Shirer et al., 2012; Belcher et al., 2013). The resulting component maps were overlaid on a high-resolution T2w squirrel monkey brain template “VALiDATe” image. Brain regions were identified and labeled by three independent observers (W.Y., F.B.M., and S.L.W.) and based on Royo et al. (2021) for cortical and Gergen and MacLean (1962) for subcortical anatomy.

The robustness of the networks was evaluated into two ways: by visually inspecting the single subject IC networks in all subjects and through cross-correlation analysis between each IC in model orders 30 with model orders 20 and 25 using FSL's “fslcc” function with default parameters and a correlation threshold of 0.1 (Table 2). The above analyses were performed for both the adolescent and adult groups, combined and separately.

### Dual regression

**Figure 2.**
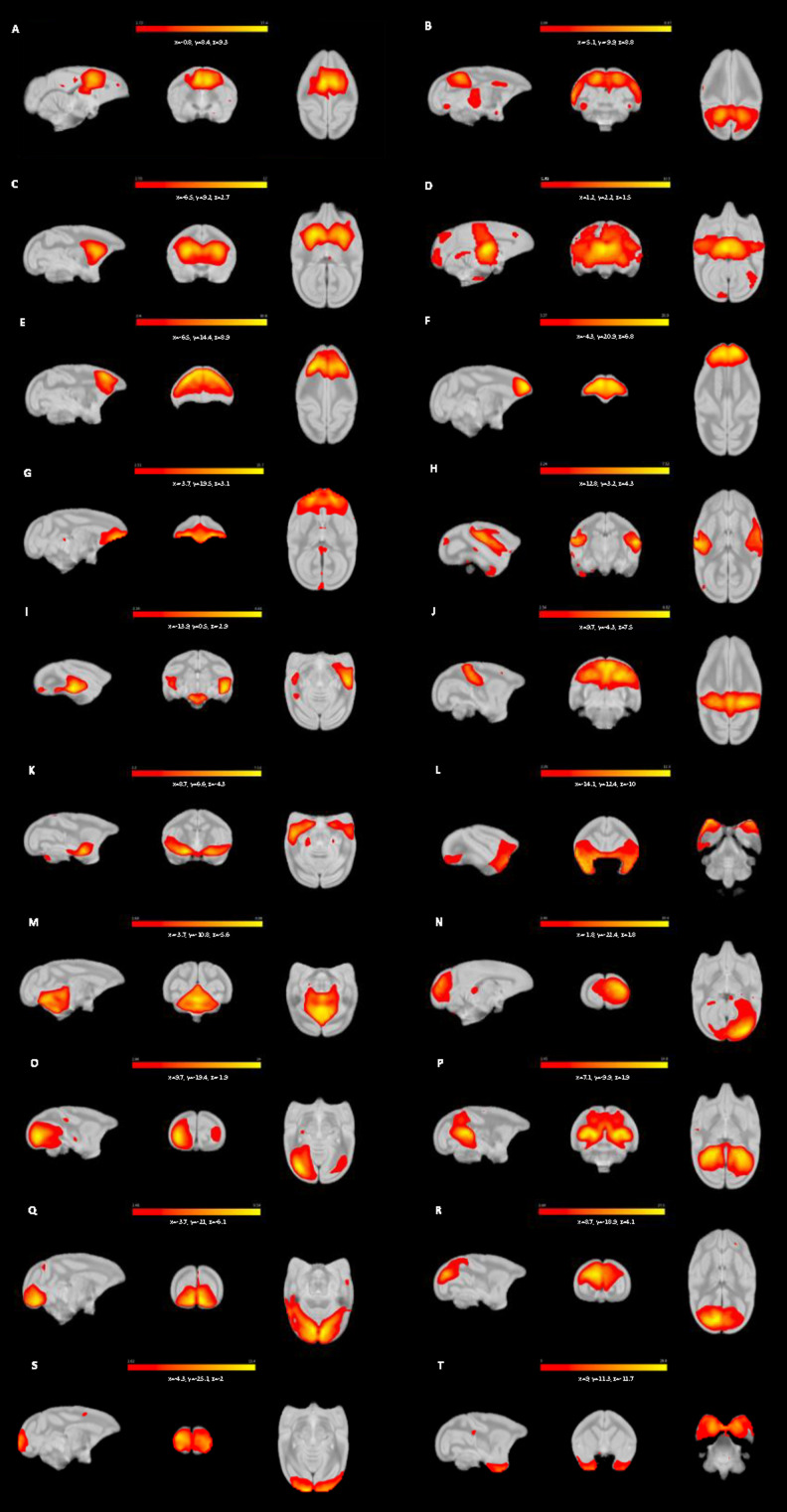
Twenty components identified as RSNs in the awake squirrel monkey (*n* = 27) using melodic group ICA with model order set to 30. Networks of similar type are presented following each other. Cingulate network (***A***), DMN (***B***), basal ganglia (***C***), thalamus (***D***), dmPFC (***E***), PFC (***F***), OFC (***G***), ventral parietal network (***H***), temporal cortex (***I***), sensory motor network (***J***), amygdala (***K*,*L***), cerebellum (***M***), visual network (***N–S***); right visual network (***N***), left visual network (***O***), V2 and V6 visual areas (***P***), V1 and V2 visual areas (***Q***), V1, V2, and V6 visual areas (***R***), primary visual network (***S***), temporal pole (***T***). Networks are shown in all three orthogonal directions overlayed on a high-resolution T2w anatomical image of VALIDATE atlas. Color bar represents *z*-scores. ICA, independent component analysis; DMN, default mode network; dmPFC, dorsomedial prefrontal cortex; PFC, prefrontal cortex; OFC, orbitofrontal cortex.

We were further interested to know whether RSNs related to higher level cognitive processing are conserved between adolescent and adult subjects and, to address this, focused on networks containing the cingulate, amygdala, PFC, insula, basal ganglia, and thalamus. This corresponded to amygdala-hippocampal network (Fig. 2*K*), amygdala temporal network (Fig. 2*L*), posterior cingulate network (Fig. 2*A*), posterior cingulate-parietal network (Fig. 2*B*), dmPFC (Fig. 2*E*), rostral PFC (Fig. 2*F*), OFC (Fig. 2*G*), basal ganglia network (Fig. 2*C*), thalamus network (Fig. 2*D*), and ventral parietal network (Fig. 2*H*). To accomplish this, subject-specific versions of the spatial maps and associated time series were generated with the spatial maps from the group average analysis using dual regression (Beckmann et al., 2009, Filippini et al., 2012). To create a set of subject-specific time series (one per group-level spatial map), for each individual subject, the group-average set of spatial maps was used as spatial regressors in a multiple regression into the subject's 4D space-time dataset. Next, those time series are used as temporal regressors, also in a multiple regression into the same 4D dataset, resulting in a set of subject-specific spatial maps (one per group-level spatial map; Nickerson et al., 2017). Then, differences between the adolescent and adult groups were assessed using Randomise in FSL (two-sample unpaired *t* test with covariates, using 10,000 permutations). This was run twice independently, once with motion and sex as covariates and another with motion, sex, and age. Clusters were produced using threshold-free cluster enhancement. The number of RSNs of interest was accounted for using Bonferroni’s correction with the original threshold set at *p* < 0.05 (FWE) and adjusted to *p* < 0.002 (FWE; Table 1).

**Table 1.** Table of statistical analyses associated with each figure

Figure	Modality	Analysis	Model order	Threshold *p* value
2	rsfMRI	Independent component analysis	20	<0.05
3	rsfMRI	Independent component analysis	25	<0.05
4	rsfMRI	Cross correlation	30:20, 30:25	*r* > 0.1
5	rsfMRI	Independent component analysis	15	<0.05
6	rsfMRI	Independent component analysis/Randomise	30	<0.05, Bonferroni’s corrected

rsfMRI, resting-state functional magnetic resonance imaging.

## Results

### Awake fMRI data quality

All subjects adapted well to awake MRI acclimation procedures and completed the 30 min scan session without incident. Heart rate and SPO_2_ remained stable and within normative ranges throughout the duration of each scan session. Mean motion during the scan sessions was 0.167 ± 0.04 mm for the adolescent subjects and 0.193 ± 0.02 mm for the adult subjects. No significant difference in motion was found between the two groups (*p* = 0.6).

### RSNs

All ICs representing white matter, cerebrospinal fluid, or physiological noise characterized as such using the methods above were not considered for further interpretation. This resulted in the identification of 20 ICs, out of the initial 30, corresponding to physiologically relevant networks that are consistent with published RSNs (Beckmann et al., 2005; Hutchison et al., 2011; Jonckers et al., 2011; Lu et al., 2012; Shirer et al., 2012; Belcher et al., 2013). These putatively labeled networks are shown in Figure 2*A–T* with additional slices of each IC shown in the supplementary material (see below) and are described as follows:

1. Posterior cingulate network (Fig. 2*A*)—This network contains the bilateral posterior cingulate cortex (PCC) and the caudate nucleus.
2. Posterior cingulate-parietal network (Fig. 2*B*)— This network includes areas that correspond to the default mode network (DMN) reported in humans (Abou-Elseoud et al., 2010) and contains the precuneus, PCC, and inferior parietal cortices.
3. Basal ganglia network (Fig. 2*C*)—This network includes the head of the caudate, globus pallidus, and putamen.
4. Thalamic network (Fig. 2*D*)—This network is localized to the thalamus with limited activation in the PCC.
5. Three PFC networks were identified—The three bilateral PFC ICs contain the dmPFC (Fig. 2*E*, which corresponds to the anterior component of the DMN; Abou-Elseoud et al., 2010), rostral PFC (Fig. 2*F*), and OFC (Fig. 2*G*) and are named accordingly.
6. Ventral-parietal network (Fig. 2*H*)—This network resembles the salience network reported in humans (Seeley et al., 2007; Seeley, 2019) and includes the bilateral ventral parietal cortex, secondary somatosensory area (PV/S2), anterior parietal cortex (APC), primary motor cortex, and premotor cortex.
7. Temporal network (Fig. 2*I*)—The temporal cortex was identified in one network localized to mid-temporal cortex and brainstem.
8. Dorsal-parietal network (Fig. 2*J*)—This network contains brain regions associated with sensorimotor networks (cf. Rocca et al., 2009). The posterior parietal network contains the bilateral APC, bilateral posterior parietal cortex, bilateral PCC, and bilateral PV/S2.
9. Amygdala-hippocampal network (Fig. 2*K*)—This network includes the bilateral amygdala and the hippocampus.
10. Amygdala-temporal network (Fig. 2*L*)—The bilateral amygdala and temporal cortex are included in this network.
11. Cerebellar network (Fig. 2*M*)—This network is limited to and covers the entire cerebellum.
12. Anterior temporal network (Fig. 2*T*)—This network contains the bilateral temporal poles.
13. The occipital cortex was found in 6 of the 20 components. There were two unilateral visual components, one right component (Fig. 2*N*) and one left component (Fig. 2*O*) each containing visual areas V1–V4. One of the six was restricted to the bilateral primary visual area (V1; Fig. 2*S*); one contained visual areas V1 and V2 (Fig. 2*Q*); one contained V1, V2, and V6 (Fig. 2*R*); and one contained V2 and V6 (Fig. 2*P*).

The RSNs identified in the group-level analysis described above were also found in the two groups (adults and adolescents) when analyzed separately. Final RSN maps and expanded view of axial slices of each network for the MO30 are available for download at https://doi.org/10.7910/DVN/RQAMYM.

### Cross-correlation

**Figure 3.**
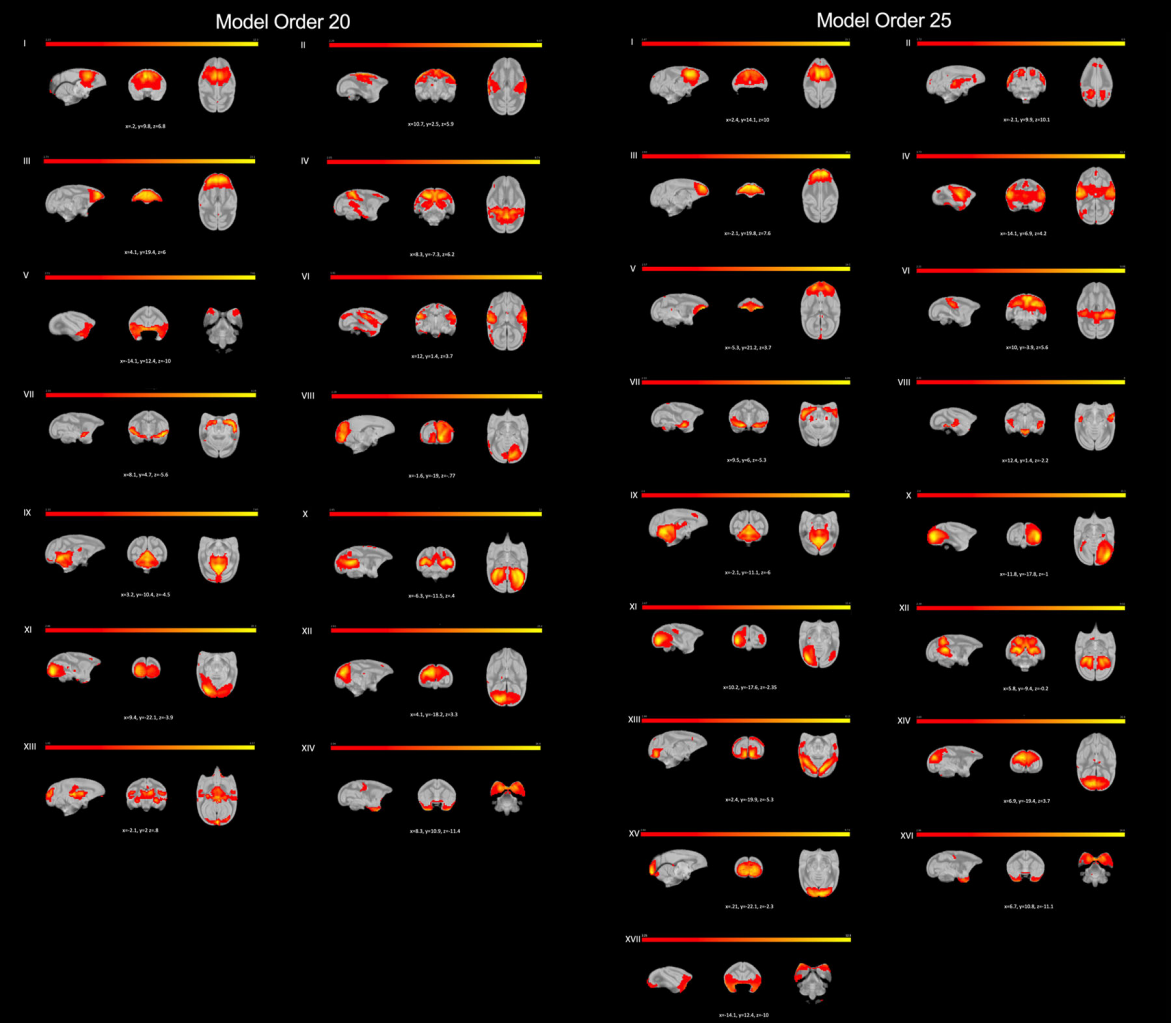
Components identified as RSNs in awake squirrel monkeys (*n* = 27) using melodic group ICA with model orders of 20 (left panels) and 25 (right panels). Correspondence of these networks with those in ICA30 (Fig. 2) are shown in Figure 4 and Table 2.

**Figure 4.**
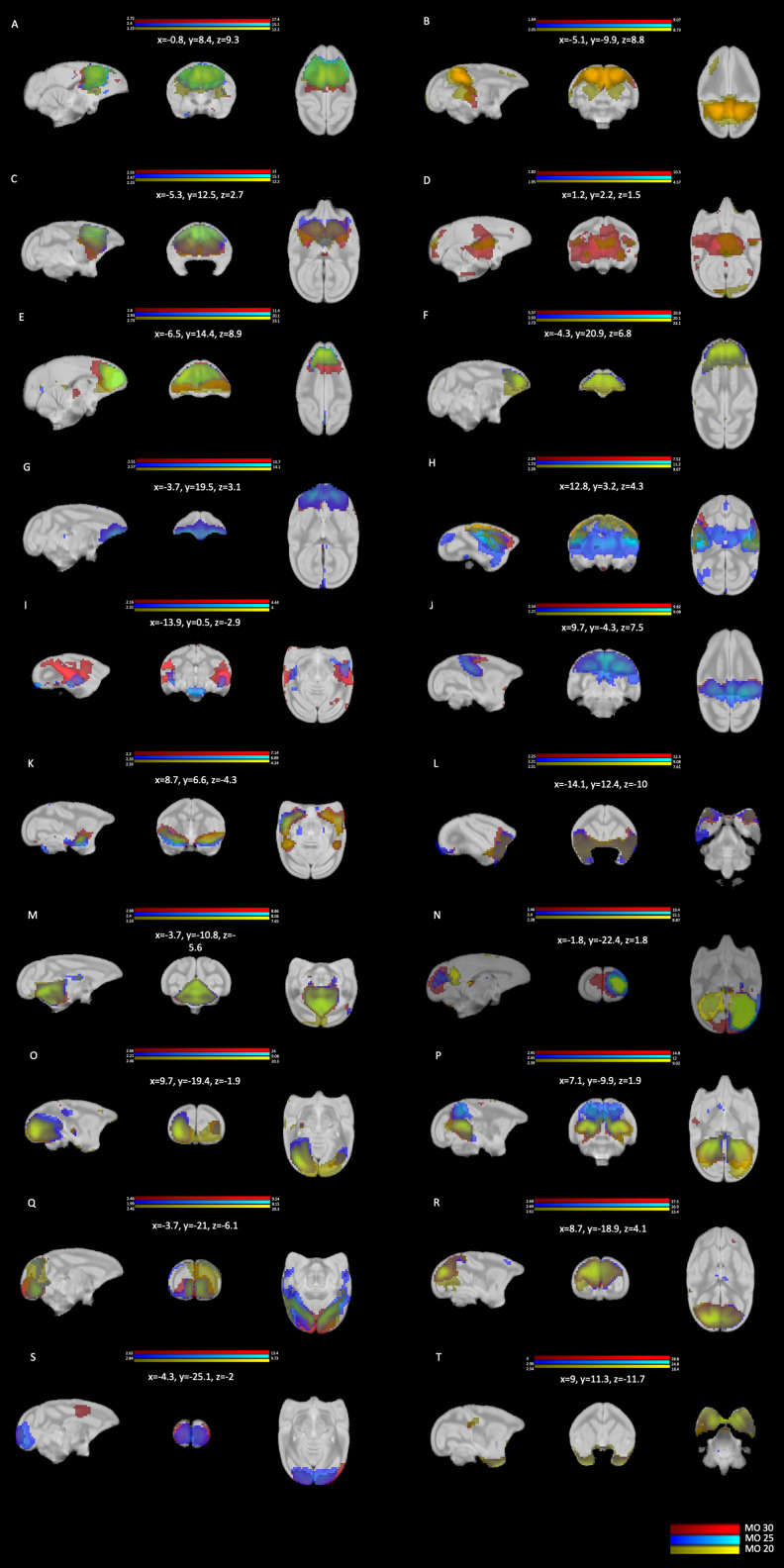
Cross-correlation results between each network in model order (MO) 30 with those in MOs 20 and 25. Spatial maps for MO30 are shown in red, MO20 in yellow, and MO25 in blue. See text and Table 2 for additional details.

Spatial cross-correlation was used to evaluate the robustness of the networks from model order 30 with those in model orders 20 and 25 shown in Figure 3; the overlap of the three model orders is shown in Figure 4 and described in Table 2. Strong correlations were found in 15 of the networks between all three of the MOs (Fig. 2*A*,*C*,*E*,*F*,*H*,*K–R*,*T*); 4 networks were not found in MO20 (Fig. 2*G*,*I*,*J*,*S*); and 2 networks were not found in MO25 (Fig. 2*B*,*D*). No unique networks were identified in either MO20 or MO25 (Fig. 4).

**Table 2.** The cross-correlation values and corresponding IC # between each network in model order (MO) 30 and MOs 20 and 25

Network ID	MO30 IC #	Network ID	MO20 IC#	Correlation (*r*)	Network ID	MO25 IC#	Correlation (*r*)
A	27	I	14	0.71	I	16	0.73
B	12	IV	3	0.81	VI; XII	9; 3	0.49; 0.48
C	23	I	14	0.44	I	16	0.36
D	25	XIII	18	0.64	II; IV	25; 18	0.47; 0.41
E	24	III; I	9; 14	0.46; 0.42	I	16	0.58
F	20	III	9	0.79	III	14	0.88
G	14	III; Noise	9; 12	0.45; 0.46	V	11	0.94
H	15	II	1	0.44	IV	18	0.53
I	28	VI	20	0.6	II	25	0.49
J	11	II	1	0.61	VI	9	0.82
K	2	VII	6	0.85	VII	5	0.8
L	10	V	5	0.82	XVII	17	0.88
M	8	IX	13	0.85	IX	13	0.89
N	3	X	2	0.5	X	2	0.83
O	7	XI	7	0.74	XI	7	0.93
P	1	X	2	0.78	XII	3	0.82
Q	6	VIII	4	0.63	XIII	15	0.7
R	5	XII	10	0.91	XIV	4	0.96
S	21	Noise	8	0.61	XV	8	0.48
T	19	XIV	16	0.84	XVI	22	0.95
Noise	4	II	1	0.57	Noise	1	0.81
Noise	9	Noise	12	0.36	Noise	12	0.4
Noise	13	Noise	17	0.93	Noise	20	0.96
Noise	16	Noise	11	0.21	Noise	10	0.86
Noise	17	Noise	12	0.65	Noise	12	0.84
Noise	18	Noise	11	0.72	Noise	19	0.92
Noise	22	Noise	15	0.72	Noise	21	0.94
Noise	26	Noise	19	0.87	Noise	23	0.94
Noise	29	VI	20	0.53	Noise	24	0.78
Noise	30	Noise; XIII	11; 18	0.34; 0.34	IV; VIII	18; 6	0.34; 0.33

Network IDs in MO 30 correspond to those in Figure 2, those in MOs 20 and 25 correspond to Figure 3. See Figure 4 for overlapped spatial maps between each MO. The correlation threshold was set at *r *≥ 0.1.

### DMN

**Figure 5.**
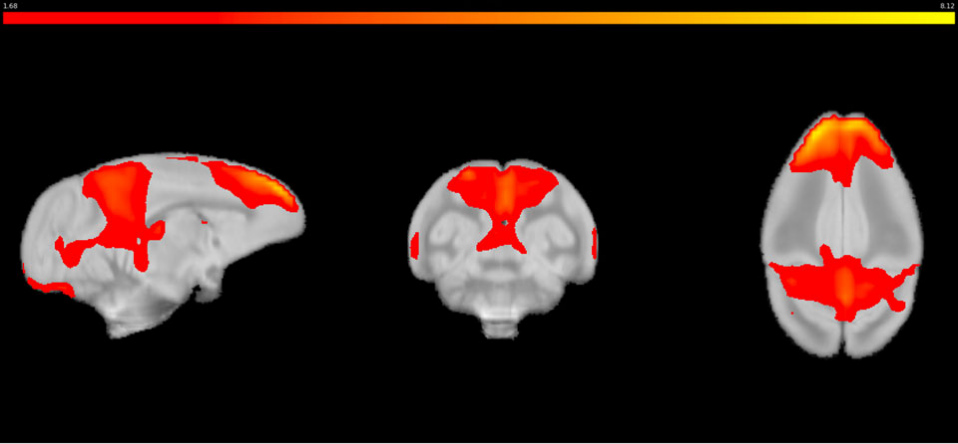
The DMN containing both anterior and posterior components found at a lower model order with an independent component number set at 15.

As the intention of this study was not to focus on a particular network per se but to identify all the potential RSNs evident in squirrel monkeys, we did not select the model order based on whether a particular network was identified. However, it was noted that in model order 30, the anterior and posterior components of DMN, considered to be a key target in neurodevelopmental and neuropsychiatric disorders (Supekar et al., 2010; Choi et al., 2021), were found in separate ICs. Given the interest in evaluating large-scale networks across species, especially DMN, lower model orders were further evaluated to determine if the posterior and anterior components of the DMN could be identified as an intact network in the squirrel monkey. Figure 5 shows the DMN observed in model order 15. While the DMN was found at model order 15, several other networks were found to overlap suggesting that this model order was not optimal for a full assessment of squirrel monkey RSNs.

### Dual regression

**Figure 6.**
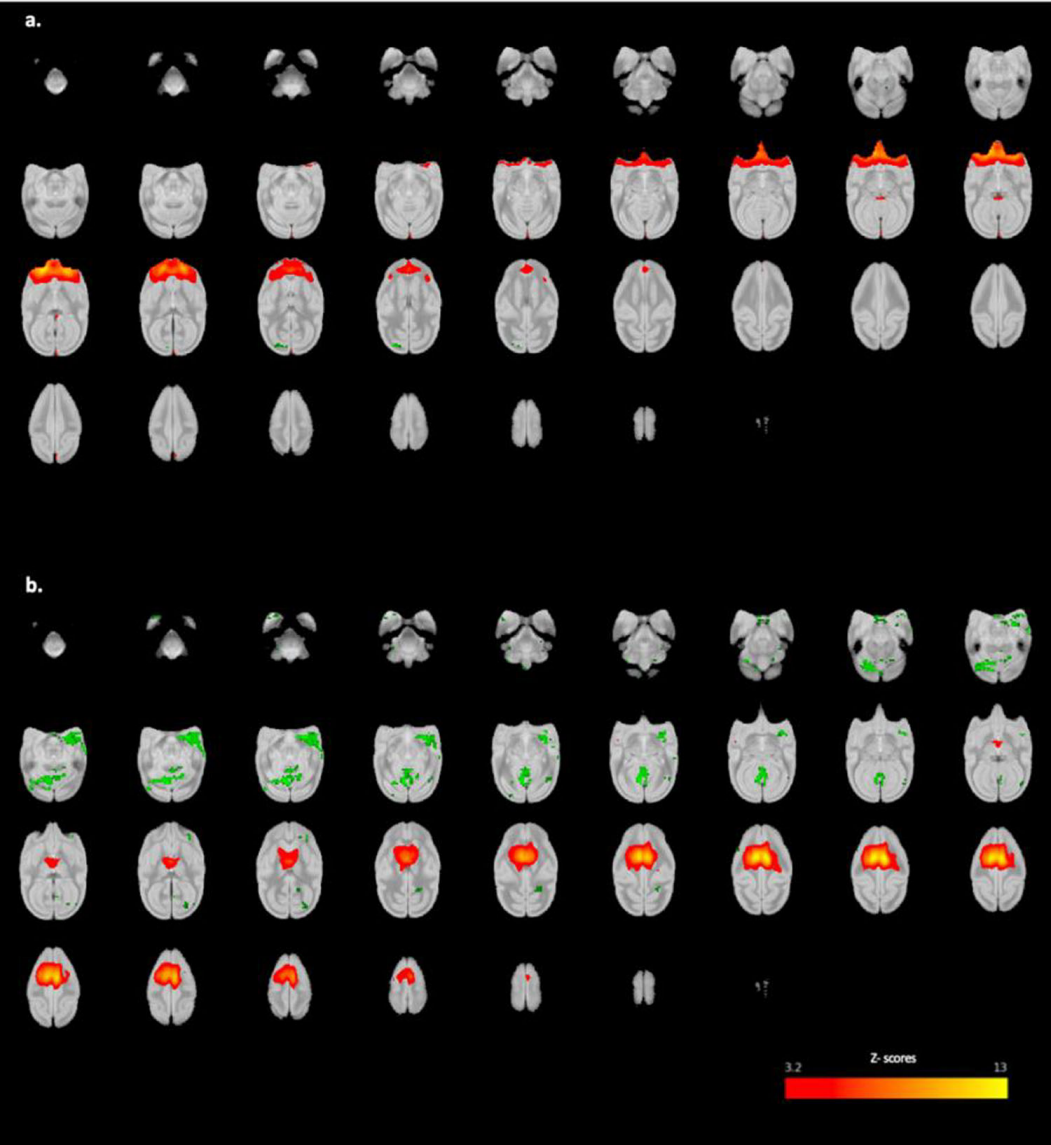
Axial brain view showing the dual regression results between adult and adolescent groups. The coordinates show dual regression results in green (adult > adolescent) where *p* < 0.003 overlayed on the corresponding IC network shown in Figure 2.

Figure 6 shows that, with motion and sex entered as covariates, adults showed a trend for greater connectivity compared with adolescent subjects in two of the networks of interest: (1) in the right occipital region (Fig. 6*A*) with the OFC network (shown in Fig. 2*G*) and (2) in the left temporal cortex, bilateral occipital cortex, and cerebellum (Fig. 6*B*) with the posterior cingulate network (shown in Fig. 2*A*); *p* < 0.003. However, when age was entered into the above model, this trend for significance was lost, even at a liberal threshold (*p* = 0.05).

## Discussion

RSNs are associated with key neurological and cognitive processes, and alterations of such networks are thought to reflect underlying pathology. As NHPs become increasingly utilized in neuroimaging studies and developments in methodology continue (see Special Issue of *Neuroimage – Nonhuman Primate Neuroimaging Is Coming of Age*, which can be found at https://www.sciencedirect.com/journal/neuroimage/special-issue/0BKLBFXR17), a comprehensive mapping of functional brain networks will facilitate their use as translational models in neuropsychiatric research—both elucidating underlying mechanisms of neuropsychiatric disorders and developing targeted treatments for their remediation. With this in mind, the goal of the present study was to characterize RSNs in both adolescent and adult squirrel monkeys at ultra-high field strength (9.4 T). Research using squirrel monkeys as experimental subjects has contributed importantly to our understanding of a range of psychiatric and neurodegenerative disease states (reviewed by Royo et al., 2021) and fills an important phylogenetic gap between rhesus macaques and marmosets; two NHP models used in a growing number of neuroimaging datasets. Furthermore, the present work was conducted in the absence of anesthetic agents—that is, subjects were awake during scan sessions—and 20 networks representing a range of neural functions, including motor, sensory, reward, and cognitive processes were identified.

The RSNs described in the current report are defined by anatomic localization and classified as large-scale networks informed by anatomical features of RSNs reported in previous studies, where appropriate (Hutchison et al., 2011; Heine et al., 2012; Belcher et al., 2013; Yacoub et al., 2020). A survey of the literature shows that the results presented here in squirrel monkeys are highly consistent with those described in a number of laboratory animals, including rodents (Moeller et al., 2009; Hutchison et al., 2010; Becerra et al., 2011; Lu et al., 2012; Bajic et al., 2017), voles (Ortiz et al., 2018), ferrets (Zhou et al., 2016), and other NHP species (Hutchison et al., 2011; Belcher et al., 2013). Importantly, most of the networks identified with ICA were consistent across several model orders suggesting robust and reproducible networks. Further, the ICs presented here contain a mixture of those that encompass both cortical and subcortical brain regions which may be probed for their association with behavioral or neurobiological functions in future studies.

One of the prominent features of RSNs in preclinical imaging studies is local connectivity rather than the long-range connections that are sometimes reported. For example, RSNs such as the DMN, which is implicated in many cognitive, social, and emotional functions (Whitfield-Gabrieli and Ford, 2012) has often been reported as being “broken” into subcomponents; the DMN in animals has been discussed in detail by Hutchison and Everling (2012); see also Liu et al. (2019) and Garin et al. (2022). The ICs containing DMN-associated regions reported here (Fig. 2*B*,*E*) do not include the full complex of structures but instead, are separated into regionally defined subcomponents (i.e., anterior and posterior). Similar partitioning of large-scale networks, including of the DMN (cf. Abou-Elseoud et al., 2010), has been reported in human fMRI data using ICA (He et al., 2009; Doucet et al., 2011). Previous investigations have identified at least two subsystems within the DMN; the dorsomedial prefrontal subsystem containing structures such as the dmPFC, temporoparietal junction, lateral temporal cortex, and temporal pole, and the medial temporal lobe system (MTL) containing brain regions including the vmPFC, posterior parietal lobule, retrosplenial cortex, parahippocampal cortex, and hippocampal formation (Andrews-Hanna et al., 2010, 2014). These dual systems are involved in various aspects of mentation and cognitive processing that interact with each other and a core set of DMN hubs (PCC and anterior medial PFC; Buckner et al., 2008; Smallwood et al., 2021). Deficits in the integration of functions across these subsystems have also been implicated in a variety of psychiatric and neurological conditions including substance use disorder (Zhang and Volkow, 2019), mood disorders (R. Liu et al., 2018), Alzheimer's disease (Jones et al., 2011), and epilepsy (Hu et al., 2017). There are two likely explanations for the partitioning of larger-scale networks among multiple ICs reported here and elsewhere: first, ICA captures unique subnetworks (i.e., dmPFC and MTL subsystems) that function dynamically over time, each with specific roles in neural processing, consistent with hierarchical functional organization of the brain. Next, the distinction may have a foundation in evolutionary biology as a recent study comparing features of the DMN in humans, macaques, marmosets, and mouse lemurs found that mPFC was weakly connected to PCC in primates when compared with humans (Garin et al., 2022). Because a full DMN emerged only at a point where several other networks also converged may suggest that anterior and posterior DMN subsystems are weakly connected (cf. Hutchison and Everling, 2012; Garin et al., 2022) in squirrel monkeys (Abou-Elseoud et al., 2010). Regardless, the fact that each of the RSNs reported here were observed in all individual subjects emphasizes the robustness of the current data and in turn, provides confidence that these networks also will be evident in future imaging studies with squirrel monkeys.

It is widely understood that the brain continues to develop from adolescence into adulthood (Blakemore, 2012; Rubia, 2013). The adult brain is believed to contain networks that consist of well-defined spatiotemporal connectivity, a reflection of increasing neural and hierarchal segregation and pruning as one ages (Stevens et al., 2009). Some studies have demonstrated that key RSNs—for example, DMN and executive network—are largely similar between children and adults; however, some studies have shown activation of frontal regions in the DMN to be weaker in children than adults, and cingulate activation was not observed in several RSNs in children (Stevens et al., 2009; Muetzel et al., 2016). In the present study, overlapping RSNs were found in both the adolescent and adult brains, which is largely consistent with findings in human subjects. However, in contrast to studies in humans, connectivity in the PFC and cingulate networks to occipital and temporal regions, respectively, was slightly greater in adult squirrel monkeys than that in adolescents. While the basis for these differences is not yet known, several factors deserve consideration. First, the human literature typically includes subjects ranging in age from early to mid-adolescence for comparisons of age-related rsfMRI whereas adolescent squirrel monkeys in the present study were a relatively homogenous group, with ages spanning only a 3 month range. Thus, it is possible that the differences between adolescents or adults identified here may reflect the well-controlled age range of the subjects studied. Second, it is possible that other types of analysis (ROI-based, graph theory, dynamic connectivity analyses) or different experimental designs (task-based fMRI) may reveal other subtle differences in brain function or connectivity that are not evident using an ICA approach. While future studies are needed to answer these questions, the results of this study demonstrate the robustness and reproducibility of the ICA networks identified here in individual subjects and across different cohorts. Presumably, these findings can serve as a platform for future studies to investigate other variables or influences on rs-functional connectivity.

Patterns of functional activation in response to various stimuli and/or treatment conditions in previous studies in squirrel monkeys (Nelson et al., 2006; Gao et al., 2016; Schilling et al., 2017; Wu et al., 2017) generally have mirrored findings from the human literature (Heine et al., 2012). For example, presentation of a noxious thermal stimulus to squirrel monkey during ultra-high field imaging resulted in fMRI responses in numerous regions (e.g., thalamus, caudate, posterior insula) that are related to nociception in humans (Wu et al., 2017). Also, repeated exposure to cocaine has been shown to alter putamen and dACC connectivity (Kohut et al., 2020), a finding that recapitulates phenomena observed clinically in humans with substance use disorders (Hu et al., 2015). These results suggest the existence of consistent functional networks in squirrel monkeys and humans. The current report further strengthens the evidence for this idea by describing RSNs in awake squirrel monkeys, that is, without the influence of anesthetic agents, using conditions that are analogous to those in human resting-state studies. Overall, the present findings indicate that squirrel monkeys can be used to characterize the functional neurobiological consequences of behavioral and pharmacological manipulations in highly controlled environmental and experimental conditions that are not possible in research with human subjects. Importantly, the capacity to conduct such research in awake squirrel monkeys without the influence of anesthesia further increases the translational validity of squirrel monkey research into RSNs and, more generally, brain functional connectivity.

Advancements in human neuroimaging can potentially help the translational aspect of work conducted in laboratory animals at high field. There are several recognizable benefits of having clinical MRI scanning systems with high-field strength such as improved spatial resolution and better overall image quality (Rutt and Lee, 1996; Vachha and Huang, 2021). Having such high-field systems available in a clinical setting may help reduce the need for major optimization methods between findings from NHP or other animal species and humans and warrant that data coming from NHPs can be more directly compared with those from human imaging modalities. This can improve both the translational aspect of preclinical research into clinical practice as well as the reproducibility of findings.

Despite the reliability, robustness, and strength of the ICA method, it is important to acknowledge its limitations, especially sensitivity to motion (Parkes et al., 2018) requiring the removal of motion-related ICs from the data. This is typically done based on the assessor's judgment and requires specific knowledge of the RSNs, especially in animal neuroimaging, or the use of more rigorous motion correction tools (Parkes et al., 2018). The uncertainty in determining the number of ICs in the ICA method is also important and, as mentioned earlier, may impact RSNs. Machine learning techniques may be useful for addressing this challenge (Wang and Li, 2015). Future studies highlighting the connectivity patterns of particular networks of interest also might benefit from implementing a seed-based analytical approach or methods such as connectome, graph theory, or dynamic ICAs to delineate different hub locations and to characterize how well these hubs are connected to different brain nodes as well as the signatures of the long and short distance connections between nodes.

## Synthesis

Reviewing Editor: Christie Fowler, University of California Irvine Decisions are customarily a result of the Reviewing Editor and the peer reviewers coming together and discussing their recommendations until a consensus is reached. When revisions are invited, a fact-based synthesis statement explaining their decision and outlining what is needed to prepare a revision will be listed below. The following reviewer(s) agreed to reveal their identity: NONE. Note: If this manuscript was transferred from JNeurosci and a decision was made to accept the manuscript without peer review, a brief statement to this effect will instead be what is listed below.

This manuscript was reviewed by two experts in the field. Both felt the studies were of interest to increase the knowledge base for the use of squirrel monkeys in neuroscience research, as well as other similar animal models. However, weaknesses were noted. With regard to the statistical reporting, it was noted as insufficient. Specifically, it is not clear how the dual regression analysis was conducted, or if only a subset of the ICs was used for comparing the two age groups. Per eNeuro policy, a statistical table should also be included. Further, it is unclear as to why only two of the figures are included in the main manuscript; the supplementary material could be integrated as part of the main manuscript. Further comments are as follows:

Reviewer 1

Resting state networks of awake adolescent and adult squirrel monkeys using ultra-high field (9.4T) functional magnetic resonance imaging.

In this paper the authors present the characterization of squirrel monkeys RSN. The employ a 9.4T scanner and ICA to identify RSN. Their results demonstrate that squirrel monkey RSNs are somewhat consistent with RSNs previously identified in humans, rodents, and other nonhuman primate species. They show variability on RSN with maturation on a subset of RSNs potentially describing developmental circuits of squirrel monkeys. In the revision, the authors have attempted to address reviewer concerns; however, some answers were a bit underwhelming. Overall, this is a nice article introducing and validating some RSN in another animal model and it provides a medium advancement in the field, however there are some concerns regarding the handling and interpretation of some of the results.

I agree with the authors that awake imaging is more appropriate, however, most of the literature on animals in done with anesthesia and there is work implying how minimize the effect of anesthesia. One issue with awake and the training it entails is the notion of learned helplessness. Is there a measure of stress on these monkeys to show that they are in fact trained and not in a different state? Is there an awake vs a reasonable anesthetic regimen comparison to show that they are different?

The IC is done with 30 components. I agree with the reviewers that we must start somewhere, and they made a reasonable decision. However, a comparison on a few components would show robustness of their results. Particularly with the fragmented nature of the DMN in squirrel monkeys, which they tried to resolve by reducing the components to 15 and finding a complete DMN however, it came with a large overlap to other networks. This a larger issue that is unresolved and could be interpreted two ways: squirrel monkeys RSN are a bit different that humans (not unreasonable and interesting as an experimental question going forward) or the results are just a consequence of experimental decisions which makes the work less interesting and more problematic. I believe they need to add their results for 20 and 25 and show correspondence and preservation of the RSN. 15 ICA components may be small number and unsurprising that there is a large overlap. The authors want this paper as a resource to the field, but it is unclear whether they are showing significant RSNs or a random sampling of ICA components. I am leaning towards the former, but they need to show their results to support their claims.

Also, is there data on the motion of squirrel monkeys during scanning? How stringent is the helmet?

Overall, this is an interesting and potentially helpful reference with some additional work. The methods are straightforward and easily replicable with the caveat that the 30 IC are the best set up parameter for this species.

Reviewer 2

This paper is an important step forward in developing imaging methodology in squirrel monkeys, and is technically impressive. I have some questions re the approach, and comments on data analysis/interpretation.

Was a single helmet used for all subjects?

What are the normative ranges for heart rate and spO2 mentioned in the first paragraph of the Results, and is it correct that those bounds were never exceeded in any scan?

On what basis were ten out of thirty ICs "representing white matter, CSF, or physiological noise" excluded? Was it a necessity that they were entirely anatomically within white matter or the ventricles, or just a subjective call that they seemed too much within those bounds? How was a "physiological noise" network identified?

The authors state that 20 physiologically relevant networks is consistent with published results, they cite (among others) Hutchison et al., 2011, but that study just identified 11 networks in macaques. This discrepancy is emblematic of a general concern that the identities of RSNs (resting state networks) and their bounds are highly dependent on analysis approach which leads me to the next paragraph.

There has been substantial discussion in the literature re whether a nonhuman primate (macaque) DMN (default mode network) contains a dorsomedial prefrontal cortical component (as summarized in the Hutchison and Everling, 2012 and Garin et al., 2022 refs). In general, investigators need to bend over backwards to demonstrate it, often by playing around with seed locations to show connectivity of components, or in the present case, by manipulating the model order to 15, at which point now an "intact DMN" emerges that looks more like that seen in humans. This approach is extremely subjective, and in my mind, confers little useful information as to the bounds of a RSN analogous to the DMN of humans. Also, at this point the authors note (line 215) that the lower model order results in overlap of several networks, suggesting it is less than optimal. In this regard, I have issue with the following section of the Discussion: "Although the ICs containing DMN-associated regions reported here (Figs 1E and 1B) do not include this full complex of structures, the overall pattern of connectivity identified over multiple ICs strongly suggests evidence of a squirrel monkey DMN ortholog (cf. Hutchison and Everling, 2012; Garin et al, 2022), albeit separated into regionally defined subcomponents (i.e., anterior and posterior)." If an "intact DMN" can only be revealed by manipulating the analysis/interpretation, it appears that is being done to achieve a desired result. My recommendation is to tone down this interpretation.

The Dual regression approach and results are inadequately described. The Beckmann et al, 2009 reference they cite as a reference for the method is a poster title, nothing more, and so is of no use. In the Results, it is stated (line 219) that in looking for differences between the adolescent and adult groups, they would focus on "networks containing cingulate, amygdala, PFC, insula, basal ganglia, and thalamus." Does this mean they are only looking at a subset of networks? Is their Bonferroni corrected threshold p < 0.002 for significance as indicated on line 160? If so, isn't their p value of 0.003 greater than their threshold? I'm inclined to agree with Reviewer #3 that the group difference is a bit random, and a lack of one would not diminish the paper.

The authors refer to a volume of Neuroimage (244) without any accompanying reference to address a previous comment about the lack of references to recent important literature on developments in NHP imaging. Perhaps the authors are referring to "Article(s) from the Special Issue on Nonhuman Primate Neuroimaging is Coming of Age; Edited by Sabine Kastner, Daniel Margulies, Charlie Schroeder, Michael Milham and Chris Petkov." If so, that "Special Issue" is actually 30 articles spread out over 13 volumes, of which #244 only has one, "Dynamic reconfiguration of macaque brain networks during natural vision." One can link to the collected set of papers from the journal, but I'm not sure how to best reference this formally.

Do the authors really mean to use "orthogonal" in line 308? I don't understand if so.

Extended Data:

Need to clarify the value in the lower order IC analysis done in order to reveal an "intact DMN."
